# Internet-based cognitive behavioral therapy for individuals with gambling disorder in Indonesia: protocol for a pilot and feasibility study

**DOI:** 10.12688/f1000research.151009.1

**Published:** 2024-06-25

**Authors:** Kristiana Siste, Enjeline Hanafi, Belinda Julivia Murtani, Michael Baigent, Ben J Riley, Jayne Sessions, Lee Thung Sen, Hans Christian, Astria Aryani, Kevin Surya Kusuma

**Affiliations:** 1 Department of Psychiatry, Faculty of Medicine, Universitas Indonesia, Jakarta, Indonesia; 2 Department of Psychiatry, College of Medicine and Public Health, Flinders University and Flinders Centre for Gambling Research, Statewide Gambling Therapy Service, Flinders Medical Centre, Flinders University, Adelaide, South Australia, Australia

**Keywords:** gambling disorder, internet based, cognitive behavioral therapy, treatment, Indonesia

## Abstract

**Background:**

Gambling disorder (GD) has become a wide concern in Indonesia, as many negative consequences arise from this psychiatric condition. Prompt treatment with an appropriate method of delivery is required to achieve optimal outcomes in GD patients. This protocol paper outlines a study to determine the effectiveness, acceptability, and feasibility of internet-based cognitive behavioral therapy (iCBT) in treating GD in Indonesia.

**Methods:**

This non-randomized pilot and feasibility study will recruit 20 people with GD. All participants will receive the iCBT intervention through self-learning videos and guided weekly group sessions. The effectiveness of the intervention will be assessed at baseline (week 0), post- treatment completion (week 10), and 6 weeks post-treatment (week 16). The outcomes measured will be the change in gambling symptoms, gambling urges, cognitive distortions, readiness to change, emotional problems, and quality of life of the participants.

**Discussion:**

The feasibility of iCBT for GD patients in Indonesia will be assessed by this study. The study's results will give an indication of the acceptability of the intervention and the feasibility of a subsequent conclusive trial. The delivery of iCBT may help to address the issue of treatment access in an extensive geographical region and provide immediate implications as a treatment resource for GD in practice.

## Introduction

According to the Diagnostic and Statistical Manual of Mental Disorders (DSM-5), gambling disorder is defined as a persistent and recurrent problematic gambling behavior that results in substantial clinical impairment or distress. ^1^ The issue of gambling disorder (GD) is growing worldwide. In recent years, more people have access to both offline and online gambling. In addition, the COVID-19 pandemic corresponded with an increase in online gambling platforms. ^3^^,^^4^ A study in Indonesia during the pandemic found the prevalence of GD in Indonesia is estimated to be 1% in 2021. ^2^ This is an enormous figure as Indonesia comprised up to 278 million people. ^3^Low public awareness and a lack of effective regulation could eventually cause the number of individuals experiencing GD to rise in the future. ^3^^,^^5^

Gambling disorder causes major impacts in life including unemployment, interpersonal problems, financial difficulties, and an increased risk of other mental disorders such as depression, anxiety disorders, stress-related and somatoform disorders, along with substance use disorder. ^2^ Therefore, it is essential to provide suitable treatment for gambling disorders. Early intervention and appropriate management have been shown to significantly improve outcomes for individuals with gambling disorders. ^6^^,^^7^

The goal of GD treatment is symptom remission and functional recovery. To date, no pharmacological treatment has been approved for GD. However, some studies have demonstrated positive outcomes when pharmacological and psychotherapy treatments are combined. ^8^ Cognitive behavioral therapy (CBT) is an effective treatment modality for GD. ^8^^,^^9^ Studies reveal that CBT reduces gambling-related symptoms and problematic behavior whilst also improving the overall functioning and quality of life of people with GD. ^7^^,^^9^ In recent years, internet-based cognitive behavioral therapy (iCBT) has been suggested as a means of lowering barriers to professional help seeking by showing equally promising outcomes with increased accessibility, convenience, and cost-effectiveness. ^10^^–^^12^ Studies of help-seeking among individuals with gambling problems consistently report very few (less than 10%) gamblers seek formal help. ^13^^–^^15^ Similar findings also come from an Indonesian study that indicated only 15.5% of people seek treatment for GD. ^2^ Problem gambling and its consequences can remain hidden within families, usually over extended periods, and help-seeking is often the last resort after significant adverse events or crises such as family breakdown, loss of employment, legal issues, or deterioration of mental health. ^16^^,^^17^ Since delivering treatment for gambling disorders is challenging in Indonesia due to its vast and varied territory, iCBT appears to be a promising treatment option. However, research regarding the effectiveness of iCBT is still very scarce in Indonesia. This pilot study aims to examine the acceptability and feasibility of iCBT for GD in Indonesia.

PROTOCOL

## Methods

### Study design

**Figure 1.**
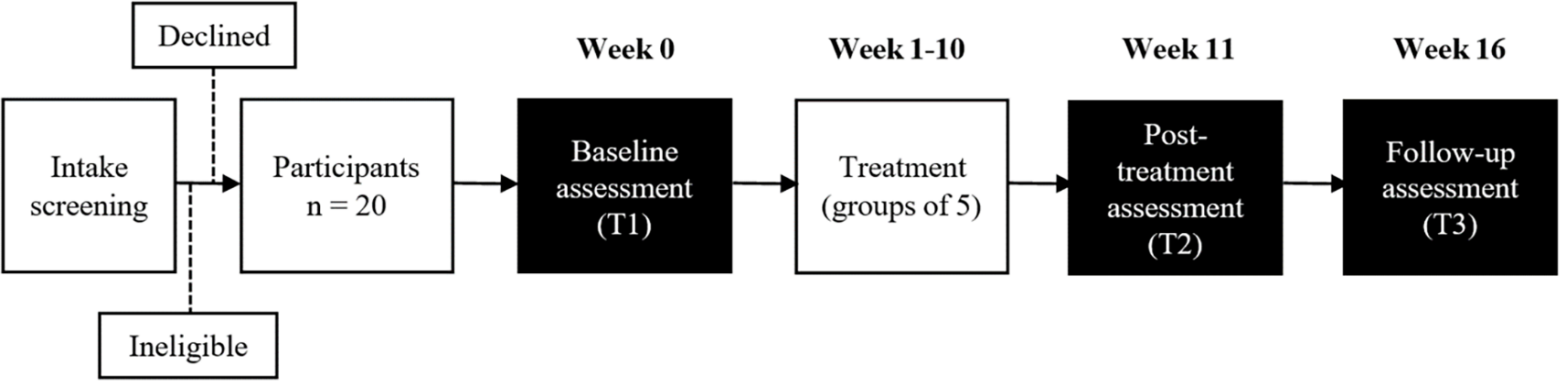
Study flowchart.

This study is a pilot and feasibility study with a quasi-experimental design. The protocol adheres to the Standard Protocol Items: Recommendations for Interventional Trials (SPIRIT) checklist (Extended data: Supplementary file 1). ^18^ After intake screening, participants will undergo baseline assessment (T1) and will be given treatment for 10 weeks. This will be followed by follow up assessment immediately after treatment (T2) and follow up assessment 6 weeks after treatment (T3) ( Figure 1).

### Participants and settings

A total of 20 participants will be recruited using online advertisements via social media and through consecutive sampling by directly approaching current and former patients in inpatient and outpatient services in Cipto Mangunkusumo Hospital, Indonesia.

The inclusion criteria include age between 18-59 years old, fluent in Bahasa Indonesia, be screened as probable pathological gambler (indicating severe and pervasive problem gambling) based on South Oaks Gambling Screen (SOGS), and do not have comorbid severe psychiatric disorder (i.e., psychotic disorders, severe mood disorders, and intellectual disability). Exclusion criteria are no history of gambling in the past six months, history of neuropsychiatric disorders (e.g., seizures), and inability to access web browsers and online conferencing platforms. Drop-outs are defined as those who miss three consecutive sessions of the 10-week therapy program for any reason.

The South Oaks Gambling Screen is chosen as a screening tool because of its widespread use internationally. ^19^ The adaptation to Indonesian version in community setting yielded a good validity and reliability testing, indicating SOGS as a good instrument to screen for GD in Indonesia. ^2^

Participant eligibility will be assessed by medical doctors at Dr. Cipto Mangunkusumo General Hospital, Jakarta, Indonesia. A psychiatric assessment utilizing the ICD-11 criteria for gambling disorders will be carried out to confirm the patient's diagnosis of GD.

**Figure 2.**
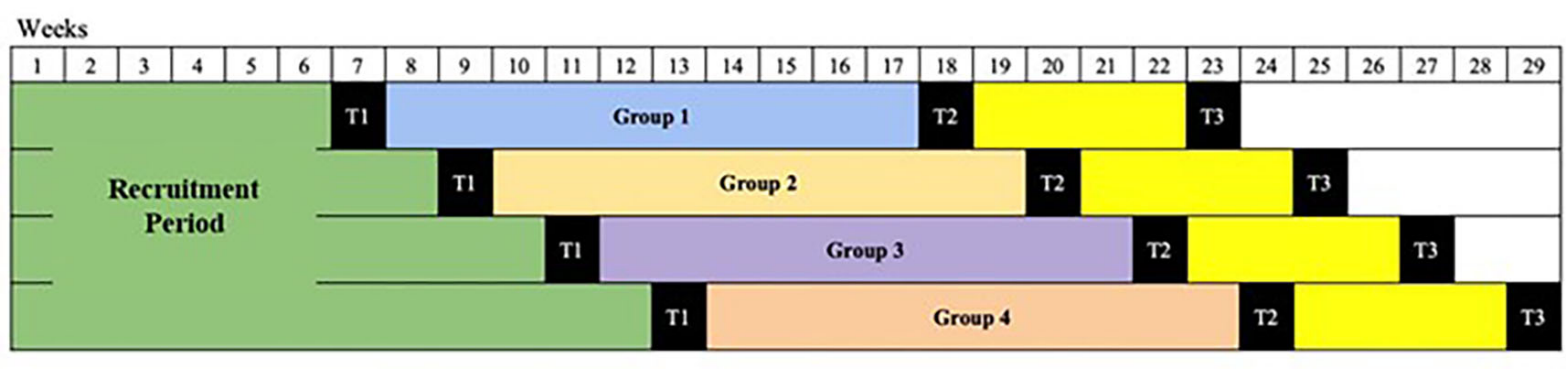
Study timeline.

Following the determination of a participant’s eligibility for the study, the Indonesian version of Gambling Symptom Assessment Scale (G-SAS), Gambling Urge Scale (GUS), Gambling Related Cognitions Scale (GRCS), and Barratt Impulsiveness Scale-11 (BIS-11) will be administered to evaluate the participants' symptoms, gambling urges, cognitive distortions and impulsivity, respectively. The University of Rhode Island Change Assessment Scale (URICA) will be used to evaluate the participants’ readiness to change. The World Health Organization Quality-of-Life Scale (WHOQOL-BREF) will be used to evaluate quality of life and the Self Rating Questionnaire-20 (SRQ-20) will be used to assess emotional problems. ^20^^–^^30^ In order to evaluate the treatment outcome, a direct psychiatric assessment of GD using ICD-11 criteria will also be conducted at the end of treatment. The assessment schedule is presented in Table 1 and Figure 2.

**Table 1.** Outcome and measurement.

Outcome	Measurement	Data for analysis	Type and score range	Hypothesis	Assessment time point		
					T1	T2	T3
Psychiatric clinical interview	Gambling disorder criteria based on ICD-11	Number of criteria	Categorical, GD or no GD	Lower	v		v
Gambling symptom severity	G-SAS	Sum of 12 items	Continuous, 8 (mild) to 48 (extreme)	Lower	v	v	v
Gambling urges	GUS	Sum of 6 items	Continuous, 0 to 42, the higher the score indicating the higher the urges/craving	Lower	v	v	v
Gambling- related cognitive distortions	GRCS	5 domains, with the highest percentage being the most dominant domain.	Continuous, 0% to 100% per domain	Lower	v	v	v
Impulsivity	BIS_11	Sum of 30 items	Continuous, 30 to 120, the higher the score indicating the higher levels of impulsivity	Lower	v	v	v
Readiness to change	URICA	Means of each stage of change score	Continuous, means of 9.3 (pre-contemplation), 11 (contemplation) and 12.6 (participation)	Lower	v	v	v
Nonspecific psychological distress	SRQ-20	Sum of 20 items	Continuous, 0 to 20, with scores >5 indicating mental distress	Lower	v	v	v
Quality of life	WHOQOL- BREF	4 domains, with transformed score used	Continuous, 0 to 100 per domain, the higher the score the higher the quality of life	Higher	v	v	v

G-SAS, Gambling Symptom Assessment Scale; PGSI, Problem Gambling Severity Index; GUS, Gambling Urge Scale; GRCS, Gambling Related Cognitions Scale.

### Development of the iCBT

The iCBT module was developed by researchers from Department of Psychiatry, Faculty of Medicine, Universitas Indonesia and Department of Psychiatry, Flinders Centre for Gambling Research, South Australia. This module consists of 6 parts which will be used as a guide for the therapist in the therapy sessions. The module content for each session is described in Table 2.

**Table 2.** Description of iCBT module content.

Session	Module content
1	Building rapport and assessment
2	Psychoeducation and objectives
3	Understanding gambling behavior
	Exposure therapy (first exposure task)
4	Identification of gambling thoughts and cravings
	Review of first exposure therapy task and planning of next exposure task
5-9	Exposure therapy
10	Relapse and prevention strategies

The contents of iCBT module are largely derived from The Flinders/Statewide Gambling Therapy Service (SGTS) Model of Treatment for Gambling Disorders, a manualized CBT treatment program. ^31^^,^^32^ The beginning of the program addresses topics such as the severity of gambling, the level of motivation, comorbidities, money management, and support systems. The intervention approach then provides psychoeducation and outlines objectives of the therapy for the participants and their significant others, aiding them with the understanding of gambling behavior, and how to identify distorted gambling thoughts and correct them. Exposure therapy including guided imagery is introduced and used to extinguish urges. Relapse prevention strategies are also included with ways to implement a balanced lifestyle, managing lapses and relapses, and ongoing money management.

### Delivering the intervention

All participants will undergo 10 therapy sessions (once-weekly) with each session lasting for 40-60 minutes. They will be divided into four small groups, each comprising five participants and a therapist. The therapy module will be given as an online self-learning source via videos in a website specifically developed for this training. The videos will divide the module content into several parts with each video lasting about 10 minutes. To confirm the participants have watched the videos, there will be a post-test measurement with no passing mark as the purpose is to assert the important points contained in the videos. The participants will then be encouraged to discuss things they understand or do not understand from their online self- learning sessions during each therapy session. There will also be several tasks that must be completed by the participants and then discussed with the therapist during each therapy session (e.g., ‘please identify the urges you feel over the next week’). Participants who miss three consecutive sessions over 10 weeks will be considered dropped out. Researchers from Flinders Centre for Gambling Research and Universitas Indonesia will meet online every two weeks to discuss participants and to provide input on treatment.

### Providers of iCBT

Two psychiatrists with expertise in GD from Department of Psychiatry, Faculty of Medicine, Universitas Indonesia will facilitate each group session in this study. The roles of the therapist are to: (1) lead and moderate iCBT sessions based on (but not limited to) the module, (2) establish a safe and warm environment, (3) provide consultation, including out-of-session, and (4) contact absent participants to encourage attendance.

### Participant characteristics

The following descriptive data will be obtained via a self-administered questionnaire: age, gender, education level, ethnicity, religion, approximate residential location, marital status, employment status, monthly income, last gambling session, age during first instance of gambling, frequency of gambling in a month, first person to introduce gambling, other people around the patient who also have gambling problems, media used to gamble, types of device used, types of gambling played, average duration of gambling during weekday and weekend, lowest and highest bet ever placed, total money lost to gambling, purpose of gambling, negative impacts from gambling, help seeking behaviors, substance use history, usage of substance during gambling session, and (if ever) rehabilitation due to substance use history.

### Sample size

The sample size for this study was determined using the formula for one sample, continuous outcome study: = (Z ∗ σ/E) ^2^, where Z is the value from the standard normal distribution reflecting the confidence level that will be used (Z = 1.96 for 95%), σ is the standard deviation score, which is 10.46 from a previous study. ^33^ Margin of error was determined to be 5 units. The sample size needed using the above formula is 17 samples, which is then rounded to 20 samples to accommodate the group therapy sessions.

### Statistical analysis

Evaluation of the response to CBT will be measured using repeated measures ANOVA. Statistical analysis will be carried out using the Statistical Package for the Social Sciences (SPSS) software version 23.0. The raw data obtained in this study only be accessible to the authors. Missing data will be handled on whether they are at random.

### Data monitoring

Data on adverse events, including hospitalization, arrest, and death, will be collected from the participants’ contact person, treating physician or medical staff. In addition, participants will be interviewed at T2 to determine whether they have experienced any subjective harmful effects (e.g., withdrawal syndrome, increased cravings) after joining this program.

### Study status

At the time of submission, the study had not begun any recruitment.

### Dissemination

The results of this study will be disseminated via peer-reviewed journals and international academic conferences. All procedures of this study will be independent from the sponsor. Participant-level dataset will not be made publicly available. Any authors included in the publications will be determined according to authorship guideline of the International Committee of Medical Journal Editors (ICMJE).

## Discussion

The aim of our study is to evaluate the feasibility of an online CBT intervention for patients with GD in Indonesia. Previous studies found that only few of those with gambling problems seek professional help. ^2^^,^^13^^–^^15^^,^^26^ Multidimensional factors including social, cultural, and personal could be contributing to their reluctance to seek treatment. There are a number of obstacles that inhibit help-seeking such as pressure from others to continue gambling, the desire to handle the issues on their own, stigma, secrecy, shame, not admitting problems, practical concerns (such as lack of availability and cost of treatment), worries about treatment, uncertainty, and avoidance. ^34^^–^^36^ Gambling is strictly prohibited under the Indonesian Criminal Code and engaging in such activities is punishable by law. ^37^ Although there is currently no research on the subject, it is possible that the illegality of GD is one of the contributing factors to the low rate of help-seeking in GD. Internet-based therapies have the potential to overcome these obstacles through the following means: (i) fostering a greater sense of anonymity; (ii) promoting transparency and honesty; and (iii) overcoming practical barriers like travel time to treatment facilities, conflicts between treatment and other time-constrained obligations like childcare or paid work, and (iv) provision of treatment relevant to cultural or language needs. ^38^

To our knowledge, our study will be the first to examine the effectiveness and feasibility of a CBT program which will be delivered online for patients with GD in Indonesia. The outcome of this study will provide valuable data for the development of iCBT for individuals with addiction, particularly GD in LMIC. This established module may be a beneficial addition to the resources of treatment for GD in practice.

Evidence-based treatments for GD in Indonesia and LMICs in general remain sparse. The proposed study may provide an alternative to conventional CBT when physical accessibility is an issue (as many experienced during the COVID-19 pandemic), or when the patients have limited access to professional help locally. As a result of this study's findings, iCBT might be applied in a more widespread context and utilized as a module to assist psychiatrists in engaging and treating more GD patients throughout Indonesia.

### Ethical consideration

The study protocol was approved by the Research Ethical Committee of Faculty of Medicine, Universitas Indonesia (approval number: KET-992/UN2.F1/ETIK/PPM.00.02/2023) which was issued on 24 ^th^ of July 2023. The study protocol was registered at ClinicalTrials.gov (NCT number: NCT06171516 on 14 ^th^ December 2023). The study will be conducted in accordance with the Declaration of Helsinki 1969, revision 2013.

All participants will be provided with and asked to sign a patient information and consent form (PICF). The results will be submitted for publication after finalizing the study. Personal data will be protected by separating the study data from the participants’ identifiable information and importantly, written agreement will be obtained from participants to never share others’ information with any third party even after the therapy is finished.

## Contributors

KS and EH conceptualised the study. KS, EH and BJM are the main developers of the iCBT module. MB, BJR, and JS reviewed the iCBT module. LTS and HC helped in designing study methodology. BJM, LTS, HC, AA, and KSK wrote the protocol and initial manuscript. KS, MB, BJR and JS reviewed and edited the final manuscript. KS supervised the whole study and procured grants. All authors have read and approved the final manuscript.

## Ethics and dissemination

The study protocol was reviewed and approved by the Research Ethical Committee of Universitas Indonesia on 24 ^th^ of July 2023 (approval number: KET-992/UN2.F1/ETIK/PPM.00.02/2023). All participants will be provided with and asked to sign a patient information and consent form (PICF). The study will adhere to the Declaration of Helsinki 1964, revision 2013. The results will be submitted for publication after finalizing the study.

## Trial Registration

The trial is registered on the 14 ^th^ of December 2023 at the ClinicalTrials.gov database with the identifier number NCT06171516.

## Data Availability

No data is associated with this protocol article. *Reporting guideline* OSF: Internet-based cognitive behavioral therapy for individuals with gambling disorder in Indonesia: protocol for a pilot and feasibility study, https://doi.org/10.17605/OSF.IO/JB7F5. ^39^ Licensed under CC-BY 4.0 International. The project includes:
• Spirit checklist
