# Supplementary material for: Internet-based cognitive behavioral therapy for individuals with gambling disorder in Indonesia: protocol for a pilot and feasibility study
Source: F1000Res. 2024 Sep 23;13:689. Originally published 2024 Jun 25. [Version 2] doi: 10.12688/f1000research.151009.2 (PMC11474144; doi:10.12688/f1000research.151009.2)
Supplement: Peer review history

# Reviewer report: Reviewer response for version 2

**DOI:** 10.5256/f1000research.172058.r326139

**Published:** 2024-10-14

**Authors:** Simone N Rodda

**Affiliations:** 1 Department of Psychology and Neuroscience, Auckland University of Technology, Auckland, Auckland, New Zealand

Thank you for addressing my comments. Article looks great. Perhaps consider being clear on the primary outcomes in the abstract and remove the secondary/exploratory items. 

 Good luck with the research.

Is the study design appropriate for the research question?

Yes

Is the rationale for, and objectives of, the study clearly described?

Yes

Are sufficient details of the methods provided to allow replication by others?

Yes

Are the datasets clearly presented in a useable and accessible format?

Not applicable

Reviewer Expertise:

Internet delivered interventions, help seeking and gambling disorder.

I confirm that I have read this submission and believe that I have an appropriate level of expertise to confirm that it is of an acceptable scientific standard.

# Reviewer report: Reviewer response for version 2

**DOI:** 10.5256/f1000research.172058.r326140

**Published:** 2024-09-28

**Authors:** David C Hodgins

**Affiliations:** 1 University of Calgary, Calgary, Alberta, Canada

No further comments. Suggestions have been clearly addressed.

Is the study design appropriate for the research question?

Yes

Is the rationale for, and objectives of, the study clearly described?

Yes

Are sufficient details of the methods provided to allow replication by others?

Yes

Are the datasets clearly presented in a useable and accessible format?

Not applicable

Reviewer Expertise:

Gambling disorders, including intervention.

I confirm that I have read this submission and believe that I have an appropriate level of expertise to confirm that it is of an acceptable scientific standard.

# Reviewer report: Reviewer response for version 1

**DOI:** 10.5256/f1000research.165628.r320594

**Published:** 2024-09-06

**Authors:** David C Hodgins

**Affiliations:** 1 University of Calgary, Calgary, Alberta, Canada

This is an interesting and well written proposal.  I am very eager to hear about the results and conclusions as we have lots to learn about how to provide effective treatment. I was able to review the comments of the first reviewer, Dr. Rodda, and I concur with all her points. I have several additional queries and suggestions. 

 Design:

 First, the protocol would benefit from identifying the primary and secondary outcomes.  The goals in the abstract are threefold -assessing effectiveness, acceptability, and feasibility.  (The article text identifies only 2 aims - acceptability and feasibility - although effectiveness is implicitly assumed by the methods).  It would be helpful to identify how these goals will be assessed and evaluated as successful or unsuccessful. One primary outcome for effectiveness should be identified. The sample size calculation refers to a standard deviation, but the instrument/variable is unspecified. 

 Participants:  

 One method of recruitment is approaching current patients in inpatient and outpatient services.  Would these patients be receiving treatment for gambling disorder, which seems like an unnecessary confound? 

 Eligibility includes ICD-11 Gambling Disorder assessed by a psychiatrist. Unfortunately, no standardized instrument exists for assessing these ICD criteria.  The lack of an instrument may be problematic for reliably assessing status at follow-up.  Perhaps an assessment of DSM-5 Gambling Disorder criteria with a standardized instrument such as the NODS might be more reliable?  Will the psychiatrists that provide the treatment also be conducting the follow-up diagnostic assessments? 

 Statistical Analysis:

 The analytic plan is briefly described.  Repeated measure ANOVA is proposed. Given that there are three time points, a mixed model analyses would better handle missing data. This analytic model would also allow inclusion of a nested “group” variable.  It is unclear whether “dropouts” will be followed but they should be included if possible.  I agree with Dr. Rodda that the definition of dropout seems arbitrary given that this is a pilot study.

 Discussion:

 A minor point is that the discussion describes internet interventions as possibly fostering a sense of anonymity.  Given that one of the novel aspects of this intervention is the group sessions, this advantage is unlikely to apply here.

Is the study design appropriate for the research question?

Yes

Is the rationale for, and objectives of, the study clearly described?

Yes

Are sufficient details of the methods provided to allow replication by others?

Yes

Are the datasets clearly presented in a useable and accessible format?

Not applicable

Reviewer Expertise:

Gambling disorders, including intervention.

I confirm that I have read this submission and believe that I have an appropriate level of expertise to confirm that it is of an acceptable scientific standard, however I have significant reservations, as outlined above.

# Response

**Published:** 2024-09-17

**Authors:** Kristiana Siste

**Affiliations:** Psychiatry Department, Universitas Indonesia, Depok, West Java, Indonesia

- First, the protocol would benefit from identifying the primary and secondary outcomes.  The goals in the abstract are threefold -assessing effectiveness, acceptability, and feasibility.  (The article text identifies only 2 aims - acceptability and feasibility - although effectiveness is implicitly assumed by the methods).  It would be helpful to identify how these goals will be assessed and evaluated as successful or unsuccessful. One primary outcome for effectiveness should be identified. The sample size calculation refers to a standard deviation, but the instrument/variable is unspecified. **Answer: The primary outcomes will be GUS, G-SAS, and GRCS. The secondary outcomes will be BIS-11, WHOQOL-BREF, SRQ-20, and URICA. The primary outcome used for sample size calculation is GUS.** **Feasibility: adherence to the protocol by the participants.** **Acceptability: 5-point Likert scale for satisfaction after each iCBT session.** **Effectiveness: measurement of pre- and post- outcomes.**
- One method of recruitment is approaching current patients in inpatient and outpatient services.  Would these patients be receiving treatment for gambling disorder, which seems like an unnecessary confound? 
  Eligibility includes ICD-11 Gambling Disorder assessed by a psychiatrist. Unfortunately, no standardized instrument exists for assessing these ICD criteria.  The lack of an instrument may be problematic for reliably assessing status at follow-up.  Perhaps an assessment of DSM-5 Gambling Disorder criteria with a standardized instrument such as the NODS might be more reliable?  Will the psychiatrists that provide the treatment also be conducting the follow-up diagnostic assessments?
**Answer:** **All participants will be allowed to continue their prior treatment as indicated.** **Deleted the post-treatment icd-11 interview as no standardized instruments are available to assess this outcome. Moreover the time duration of the diagnostic criteria (12 months) make it so that it is unreasonable to reassess the participants in 10 weeks.**
- The analytic plan is briefly described.  Repeated measure ANOVA is proposed. Given that there are three time points, a mixed model analyses would better handle missing data. This analytic model would also allow inclusion of a nested “group” variable.  It is unclear whether “dropouts” will be followed but they should be included if possible.  I agree with Dr. Rodda that the definition of dropout seems arbitrary given that this is a pilot study. **Answer: Suggestion accepted, all participants' data will be analyzed whether they finish the study or not.**
- A minor point is that the discussion describes internet interventions as possibly fostering a sense of anonymity.  Given that one of the novel aspects of this intervention is the group sessions, this advantage is unlikely to apply here. **Answer:** **Group therapy design was chosen as this format better supports the purpose of this study: to provide a resource effective means of quality therapy in LMIC and geographically difficult terrains for gambling disorder. We deleted the anonymity benefits in Discussion.** **In ethical consideration, we mentioned that sharing personal information is forbidden among participants, even after the therapy sessions have concluded.**

Thank you for your invaluable feedback.

# Reviewer report: Reviewer response for version 1

**DOI:** 10.5256/f1000research.165628.r300646

**Published:** 2024-08-30

**Authors:** Simone N Rodda

**Affiliations:** 1 Department of Psychology and Neuroscience, Auckland University of Technology, Auckland, Auckland, New Zealand

Thank you for the opportunity to review this protocol for iCBT for an Indonesian cohort of people with Gambling Disorder. An interesting study and well-written protocol. Some minor points of clarification are listed below.

 Citations 13-15 are individual studies on help-seeking, but this is superseded by a meta-analysis on population prevalence studies from around the world which reported one in five people with problem gambling had sought treatment and one in 25 at moderate risk, so the suggestion that it is 10% is not entirely correct. See (Bijker R,et al., 2022 [Ref 1])

**Methods**

 Figure 1 refers to ‘groups of 5’ under treatment, suggesting the treatment is group-based, which so far hasn't been stated. Figure 2 also refers to groups – but there’s nothing in the text on it until well into the methods under delivering the intervention.

 The delivery section suggests in-person treatment, but this is not reported anywhere earlier in the introduction or aims. It is also discussed that it is group therapy with iCBT consisting of videos and activities. This is really interesting and novel but hasn’t been stated in the background, aims, or study design. I’m not sure I know of other treatments that blend in-person with iCBT – so really novel – and if not, then the previous studies should be reported. I note under sample size it is referred to as group therapy – if so, what BCTs are being delivered then and how does this relate to the iCBT content?

 It would be great to have a table of the specific BCTs being delivered, when and how frequently, and how they are being delivered (therapist, video, written, other) and then link that to the outcome measures. For instance, I see an outcome measure of impulsivity, and it would be good to know exactly which BCT is expected to change that variable, etc. I don’t think Table 2 is sufficient for replication or to be able to map intervention components to outcomes. TREND reporting guidelines state that sufficient intervention information should be provided so as to allow for replication.

 I wonder, given the intervention is exploratory, whether it is appropriate to describe participants who do not complete the entire 10 weeks as drop-out? Perhaps an alternative is to use the feasibility study as a way of determining the optimal duration/configuration of the iCBT. Looking later at the manuscript, it looks like drop-out is from in-person treatment rather than the iCBT?

 Given the ICD-11 is being used in the interview, I wonder if it is necessary to burden participants with the SOGS given it is screening only. Perhaps consider a briefer screen – there are meta-analyses on brief screens to detect GD.

**Discussion**

 Given that gambling is illegal, perhaps there needs to be a stronger case made for group therapy, which by its very nature sounds problematic for anonymity, legal and criminal issues, and chances of sharing gambling information.

 The discussion again refers to the treatment being delivered online. It would be good to be very clear on who is delivering what behaviour change technique and how (see Michie work on BCTs if not familiar).

**Minor **Instead of ‘pathological gambler’ – consider person with pathological gambling or similar.

Is the study design appropriate for the research question?

Yes

Is the rationale for, and objectives of, the study clearly described?

Yes

Are sufficient details of the methods provided to allow replication by others?

Yes

Are the datasets clearly presented in a useable and accessible format?

Not applicable

Reviewer Expertise:

Internet delivered interventions, help seeking and gambling disorder.

I confirm that I have read this submission and believe that I have an appropriate level of expertise to confirm that it is of an acceptable scientific standard, however I have significant reservations, as outlined above.

## References

[1] : Global prevalence of help-seeking for problem gambling: A systematic review and meta-analysis. *Addiction* .2022;117(12) : 10.1111/add.15952 2972-2985 10.1111/add.15952 35830876 PMC9796401

# Response

**Published:** 2024-09-17

**Authors:** Kristiana Siste

**Affiliations:** Psychiatry Department, Universitas Indonesia, Depok, West Java, Indonesia

- Citations 13-15 are individual studies on help-seeking, but this is superseded by a meta-analysis on population prevalence studies from around the world which reported one in five people with problem gambling had sought treatment and one in 25 at moderate risk, so the suggestion that it is 10% is not entirely correct. See (Bijker R,et al., 2022 [Ref 1]).

**Answer: We have revised the number of treatment-seeking gamblers according to Bijker R et al., 2022 in the last paragraph of the Introduction.**

- Figure 1 refers to ‘groups of 5’ under treatment, suggesting the treatment is group-based, which so far hasn't been stated. Figure 2 also refers to groups – but there’s nothing in the text on it until well into the methods under delivering the intervention. 

**Answer:** **We added the suggestion in the paragraph above figure 1.**

- The delivery section suggests in-person treatment, but this is not reported anywhere earlier in the introduction or aims.

**Answer: We added the suggestion in the study design section.**

- It is also discussed that it is group therapy with iCBT consisting of videos and activities. This is really interesting and novel but hasn’t been stated in the background, aims, or study design. 

**Answer: The treatment will consist of individual online learning sessions delivered through pre-recorded videos, followed by weekly online group therapy sessions with a therapist. Each group therapy session will include five participants and will occur over a 10-week period.**

- It would be great to have a table of the specific BCTs being delivered, when and how frequently, and how they are being delivered (therapist, video, written, other) and then link that to the outcome measures. For instance, I see an outcome measure of impulsivity, and it would be good to know exactly which BCT is expected to change that variable, etc. 

**Answer: We have now specified them in Table 2.**

-  Given the intervention is exploratory, whether it is appropriate to describe participants who do not complete the entire 10 weeks as drop-out? Perhaps an alternative is to use the feasibility study as a way of determining the optimal duration/configuration of the iCBT. Looking later at the manuscript, it looks like drop-out is from in-person treatment rather than the iCBT? 

​​​​​​​ **Answer:** **There will be no drop out criteria in this study. Adherence to the treatment will be evaluated to optimize the configuration of the iCBT for future studies.**

- Given the ICD-11 is being used in the interview, I wonder if it is necessary to burden participants with the SOGS given it is screening only. Perhaps consider a briefer screen – there are meta-analyses on brief screens to detect GD.

 
**Answer:** **We will be using SOGS as it is validated and proven reliable in Bahasa Indonesia.**

- Given that gambling is illegal, perhaps there needs to be a stronger case made for group therapy, which by its very nature sounds problematic for anonymity, legal and criminal issues, and chances of sharing gambling information. 

**Answer:** **Group therapy design was chosen as this format better supports the purpose of this study: to provide a resource effective means of quality therapy in LMIC and geographically difficult terrains for gambling disorder. We deleted the anonymity benefits in Discussion. In ethical consideration, we mentioned that sharing personal information is forbidden among participants, even after the therapy sessions have concluded.**

- The discussion again refers to the treatment being delivered online. It would be good to be very clear on who is delivering what behaviour change technique and how (see Michie work on BCTs if not familiar). 

**Answer: Specified in Table 2.**

- Instead of ‘pathological gambler’ – consider person with pathological gambling or similar. 

**Answer: We added the suggestion, except for “probable pathological gambler” classification in SOGS.**
